# A Cross-Machine Intelligent Fault Diagnosis Method with Small and Imbalanced Data Based on the ResFCN Deep Transfer Learning Model

**DOI:** 10.3390/s25041189

**Published:** 2025-02-15

**Authors:** Juanru Zhao, Mei Yuan, Yiwen Cui, Jin Cui

**Affiliations:** 1 School of Automation Science and Electrical Engineering, Beihang University, Beijing 100191, China; audrey_zhao@buaa.edu.cn (J.Z.); yuanm@buaa.edu.cn (M.Y.); zy2343223@buaa.edu.cn (Y.C.); 2 Ningbo Institute of Technology, Beihang University, Ningbo 315000, China

**Keywords:** deep transfer learning, small and imbalanced data, intelligent fault diagnosis, feature adaption, fully convolutional neural network

## Abstract

Intelligent fault diagnosis (IFD) for mechanical equipment based on small and imbalanced datasets has been widely studied in recent years, with transfer learning emerging as one of the most promising approaches. Existing transfer learning-based IFD methods typically use data from different operating conditions of the same equipment as the source and target domains for the transfer learning process. However, in practice, it is often challenging to find identical equipment to obtain source domain data when diagnosing faults in the target equipment. These strict assumptions pose significant limitations on the application of IFD techniques in real-world industrial settings. Furthermore, the temporal characteristics of time-series monitoring data are often inadequately considered in existing methods. In this paper, we propose a cross-machine IFD method based on a residual full convolutional neural network (ResFCN) transfer learning model, which leverages the time-series features of monitoring data. By incorporating sliding window (SW)-based data segmentation, network pretraining, and model fine-tuning, the proposed method effectively exploits fault-associated general features in the source domain and learns domain-specific patterns that better align with the target domain, ultimately achieving accurate fault diagnosis for the target equipment. We design and implement three sets of experiments using two widely used public datasets. The results demonstrate that the proposed method outperforms existing approaches in terms of fault diagnosis accuracy and robustness.

## 1. Introduction

Fault diagnosis plays a crucial role in the health management of mechanical equipment [1,2]. With the rapid development of machine learning, particularly deep learning, intelligent fault diagnosis (IFD) has become an important topic in the engineering field [3,4,5]. Data-driven IFD methods can automatically extract meaningful features from monitoring data [6,7], establishing a link between these features and the equipment’s health status. This allows users to make timely maintenance decisions [8,9], leading to reduced downtime, lower maintenance costs, and fewer failures, thus minimizing economic losses [10] while achieving a balance between equipment reliability and cost efficiency [11].

In general, achieving high-precision IFD with deep learning requires two key assumptions: a large amount of labeled data from both normal and fault conditions [12], and data for training and testing that come from the same distribution [13]. However, in practical engineering scenarios, machines typically operate under normal conditions, with limited fault data available [14,15]. In addition, variations in operating conditions and environmental factors result in differences in data distribution [16], making it difficult to create an ideal dataset for model training [17]. When the diagnostic model is trained on limited fault data, its ability to generalize is reduced, which leads to lower accuracy and poor fault identification performance [18]. As a result, IFD in real-world applications often involves small and imbalanced datasets (S&I-IFD) [19,20], where only a small number of fault samples are used to train the model for accurate fault identification.

Transfer learning has emerged as a key technique for addressing the S&I-IFD problem [21]. Qian et al. [22] proposed a transfer learning method that leverages large amounts of balanced data from the source domain to assist target domain data, which are often limited and imbalanced. The main idea of transfer learning is to make full use of the large amount of balanced data collected from the source domain to assist the target domain data [23], which include few data and are imbalanced, for training to achieve fault diagnosis. Specifically, the source and target domains have different data distributions but contain similar information about faults, and transfer learning can improve fault diagnosis accuracy by reducing the differences between the source and target domains and learning valid information. Qin et al. [24] investigated cross-domain fault diagnosis of rolling bearings and introduced a transfer learning method based on similar distribution adaptation, effectively addressing the distribution differences between the source and target domains. Additionally, Zhu et al. [25] proposed a transfer learning approach that incorporates a refined pseudo-labeling mechanism, effectively improving fault diagnosis accuracy and robustness under varying working conditions. In this paper, the data obtained in the laboratory correspond to the source domain data and the real data to be diagnosed in the industrial environment correspond to the target domain data.

In industrial environments, it is often difficult to obtain sufficient data from the same equipment under varying conditions. To address this, many studies have utilized large amounts of fault-related data collected in laboratory settings, where damage is manually induced to simulate faults. These datasets, while balanced and containing valuable diagnostic information, often differ significantly from real-world data due to variations in equipment type, sensor placement, and operating conditions. Despite these differences, the internal composition, fault types, and failure mechanisms of the equipment remain similar, making it a valuable task to explore and transfer the features and information contained in laboratory data to real-world scenarios.

**Figure 1:**
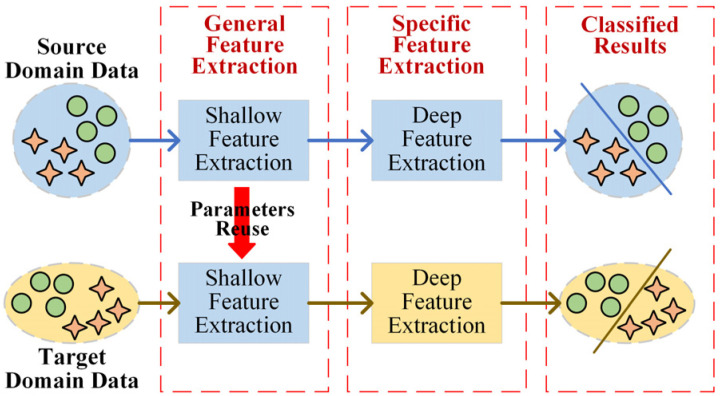
Transferability of deep learning: The shallow layer of the network is used to extract general information and the deep layer is used to extract specific information.

In recent years, deep learning has demonstrated a stronger ability for adaptive feature extraction [26], significantly reducing the complexity of feature learning. As illustrated in Figure 1, the hierarchical nature of deep learning allows the initial layers of the model to extract shallow, general features, while the deeper layers are increasingly focused on learning task-specific features [27]. This hierarchical structure makes deep learning models highly transferable. Several deep transfer learning methods have been proposed to address the S&I-IFD problem [28]. For instance, Li et al. [29] proposed a dynamic weight aggregation deep continuous transfer learning network for fault diagnosis in rotating machinery, while Wu et al. [30] introduced a multi-source domain adversarial migratory learning network, guided by conditional distribution, for fault diagnosis in rolling bearings.

Although the transfer learning methods mentioned above have addressed the issue of cross-domain fault diagnosis to some extent, they are primarily limited to cases where data are obtained from the same equipment under different operating conditions or locations. In real-world industrial settings, the target domain data for diagnosis are typically small and imbalanced, and it is often difficult to obtain data from the same equipment under alternative conditions. As a result, the aforementioned transfer learning methods have certain limitations. To overcome this, some studies have focused on generating large datasets of fault-related data in laboratory settings, where damage is intentionally induced, and then using these data for fault diagnosis. While the working conditions, size, and model of the laboratory equipment may differ from the actual equipment, the internal components, failure types, and causes of failure are often similar. Therefore, leveraging large, publicly available, and well-balanced laboratory datasets to explore the features and information they contain, and transferring this knowledge to the actual equipment, presents a valuable opportunity for enhancing intelligent fault diagnosis.

At the same time, data for fault diagnosis are usually time-series data with time-correlated properties. Unlike other types of data, there is a correlation between successive values in time-series data. Additionally, unlike two-dimensional images, time-series data contain only a single time dimension. Therefore, fault diagnosis requires the careful selection of an appropriate model and the application of methods that can effectively leverage this temporal correlation.

Additionally, fault diagnosis data are typically time-series data with strong temporal correlations. Unlike two-dimensional image data, time-series data exhibit dependencies between successive time points and contain only one time dimension. Existing methods often overlook this temporal nature, directly applying transfer strategies developed for image data. This highlights the need for methods that can effectively leverage the temporal characteristics of fault diagnosis data to enhance diagnostic accuracy.

Inspired by the above ideas, this paper proposes an S&I-IFD method based on deep transfer learning. The method leverages large and balanced equipment monitoring data obtained from laboratory settings to assist small and imbalanced monitoring data in real scenarios, building an accurate intelligent diagnosis model to achieve fault identification in real-world applications. The proposed method consists of three parts: data segmentation based on sliding window (SW), network pre-training based on residual fully convolutional networks (ResFCNs), and transfer learning. SW-based data segmentation makes full use of the temporal characteristics of the data, and the number of samples of different classes is initially balanced through the adjustment of parameters to obtain more balanced samples for subsequent feature extraction of the deep network. The ResFCN effectively extracts and mines multi-scale temporal features with the help of source domain data. Transfer learning uses a small amount of target domain data to fine-tune the pre-trained model, enabling it to learn personalized features that better match the target domain while incorporating general features from the source domain. In addition, the performance of the proposed method is evaluated using two publicly available datasets. Its effectiveness and advantages are demonstrated through a comprehensive comparison with several existing methods in related fields. The main contributions of this paper can be summarized as follows:

1. A novel method is proposed for solving the S&I-IFD problem, enabling accurate fault diagnosis in real-world scenarios by leveraging large and balanced laboratory data to assist small and imbalanced real-world data. Unlike existing methods, this work addresses the larger gap between source and target domains, making it more challenging and valuable for practical applications.
2. The method employs data segmentation techniques and network models tailored to the temporal characteristics of fault diagnosis data. It bridges the gap between source and target domain data, effectively extracting and combining the general features from the source domain with the specific features of the target domain, making full use of the available information.
3. Experiments were conducted using two publicly available datasets, and the results demonstrate the method’s effectiveness and accuracy. Two sets of comparative experiments were designed to further highlight the method’s superiority over various existing approaches.

The rest of the paper is organized as follows. Section 2 reviews related research. Section 3 presents the proposed method in detail. A series of experiments are described in Section 4, followed by a discussion of the results. Finally, conclusions are drawn in Section 5.

## 2. Related Works

### 2.1. Fully Convolutional Network

In response to the limitations of CNNs in fine-grained image segmentation, fully convolutional networks (FCNs) were proposed in 2015 to address pixel-level image classification [31]. FCNs have demonstrated impressive performance and quality. In an FCN, each output pixel serves as a classifier corresponding to the receptive field, enabling semantic segmentation based on class annotations during pixel-by-pixel training. Unlike traditional CNNs, FCNs can accept input images of arbitrary size while preserving the spatial information in the original input.

Given the capabilities of FCNs, some researchers have applied them to time-series data. For example, Park et al. [32] proposed an end-to-end arrhythmia classification system using an LSTM-FCN model, while Wang et al. [33] used an asymptotic fuzzy polynomial neural network constructed with a FCN to significantly improve the modeling accuracy of time-series datasets.

The standard structure of an FCN typically consists of a convolutional layer and a fully connected layer. The convolutional layer contains multiple convolutional kernels, each with associated weights and biases. Each neuron in the convolutional layer is connected to neurons in the previous layer within a local region, where the size of the region depends on the convolutional kernel. Assuming layer l+1 is the convolutional layer and the input is $O_{i}^{l}$, the output of the convolutional layer $O_{j}^{l+1}$ is shown in Equation (1).

(1) $$O_{j}^{l+1}(x,y)=\sigma \left(\sum_{i=1}^{I}\sum_{u=0}^{K_{1}-1}\sum_{v=0}^{K_{2}-1}O_{i}^{l}(x+u,y+v)*w_{ji}^{l+1}(u,v)+b_{j}^{l+1}\right)$$

where I is the number of input neurons at layer $l+1,\;K,\;K_{2}$ are the height and width of the convolution kernel at layer l+1, respectively, (x,y) represents the coordinates of the feature map, (u,v) represents the coordinates of the weights, $w_{ji}^{l+1}$ and $b_{j}^{l+1}$ represent the weights and biases at layer l+1, subscripts i and j represent the positions of the input neurons and the output neurons positions, respectively, and σ(.) represents the activation function.

Each node in the fully connected layer is connected to all nodes in the previous layer. Let the weight of the l−th layer be $w_{ji}^{l}$, then the output $x_{j}^{l}$ of the fully connected layer is shown in Equation (2).

(2) $$x_{j}^{l}=\sigma \left(\sum_{i=1}^{I}w_{ji}^{l}x_{i}^{l-1}+b_{j}^{l}\right)$$

where I denotes the number of input neurons of the l−th fully connected layer, $b_{j}^{l}$ denotes the bias of the l−th layer, and the subscripts i,j and σ(.) have the same meaning as the convolutional layer.

### 2.2. S&I-IFD Based on Transfer Learning

In intelligent fault diagnosis, classifier design is a crucial step as its performance directly impacts diagnostic accuracy. When training data are limited and imbalanced, classifiers often suffer from overfitting, which reduces classification accuracy. Transfer learning-based methods can improve classification performance by pre-training the classifier using data from other sources.

Parametric transfer learning is a key approach in deep neural networks, where source domain data are used to train a base model. The structure and parameters of this pre-trained model are then reused and fine-tuned with target domain data to construct a new, specialized network. This approach has been widely adopted and typically falls into two main strategies. The first strategy involves freezing all layers of the pre-trained model before the classifier and updating only the classifier parameters during training with the target domain data. For instance, Zhou et al. [34] proposed a knowledge-based U-Net and migration learning method for automatic boundary segmentation. Chen et al. [35] introduced a deep migration learning framework based on feature decomposition for concrete dam deformation prediction, while Li et al. [36] applied migration learning to predict remaining life under unknown degradation data.

## 3. Methodology

In this section, the proposed S&I-IFD model is introduced in detail, including the network structure, main components, and transfer strategy.

### 3.1. Problem Description

The proposed S&I-IFD model addresses the challenges encountered in real-world fault diagnosis scenarios. In industrial environments, data are typically imbalanced, with a larger proportion of normal data and fewer fault-related instances. As a result, intelligent diagnostic models trained on such data often suffer from underfitting or overfitting, leading to significant reductions in diagnostic accuracy. On the other hand, balanced datasets rich in fault-related information can be obtained in laboratory settings, such as through the manual induction of damage. While the internal mechanisms and failure modes of equipment in both environments are similar, differences in the working conditions, size, and model of the equipment lead to data distributions that are similar but not identical.

In this diagnostic scenario, we are given the source domain dataset $\left(X^{s},Y^{s}\right)={\left\{\left(x_{i}^{s},y_{i}^{s}\right)\right\}}_{i=1}^{n^{s}}$, where $x_{i}^{s}\in {\mathbb{R}}^{d}$ carries the class label $y_{i}^{s}\in \{1,2,\ldots ,C\}$ from the source domain $D^{s}$ and C is the number of classes. Similarly, the target domain dataset $\left(X^{t},Y^{t}\right)={\left\{\left(x_{j}^{t},y_{j}^{t}\right)\right\}}_{j=1}^{n^{t}}$, where $x_{j}^{t}\in {\mathbb{R}}^{d}$ carries the class labels $y_{j}^{t}\in \{1,2,\ldots ,C\}$ from the target domain $D^{t}$. The source domain data are balanced and the target domain data are imbalanced, that is, the target domain has more data of normal classes and fewer data of faulty classes. It is assumed that $D^{s}$ and $D^{t}$ have the same fault characteristics but follow different data distributions. In this paper, the data obtained in the laboratory correspond to the source domain data and the real data to be diagnosed in the industrial environment correspond to the target domain data. The goal of this study is to use the given data to learn the same features between the two domains, to reduce the distribution differences, and to use the learned features to predict the labels of the target domain data. In particular, the diagnostic scenario is defined as follows.

1. The equipment to be diagnosed shares the same fault categories as the laboratory equipment, with similar but not identical bearing types and operating conditions.
2. A large number of labeled samples are available for each category in the laboratory data, which can be used for the initial training of the diagnostic model.
3. The real-world training data exhibit class imbalance, with more normal data and fewer faulty instances.

### 3.2. Overall Flow of the Diagnostic Framework

The proposed method consists of three main components: sliding window (SW)-based data segmentation, which balances the data distribution and preserves temporal correlations; network pre-training using the ResFCN, which extracts general features from the source domain data; and transfer learning, which fine-tunes the pre-trained model to adapt to the specific characteristics of the target domain. This method effectively bridges the gap between the source and target domains, enabling accurate fault diagnosis even when the target domain data are small and imbalanced.

**Figure 2:**
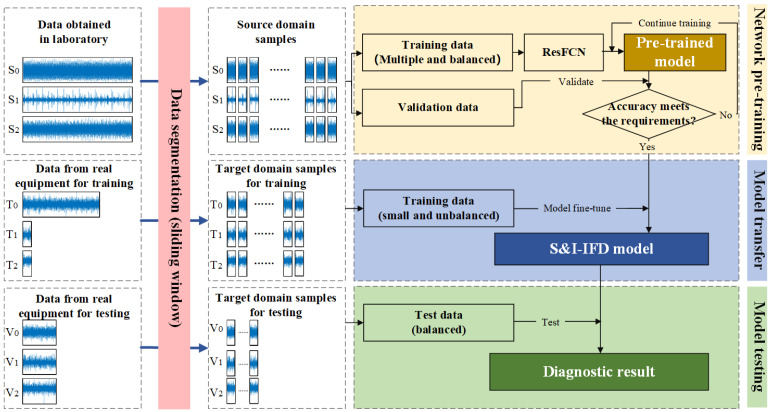
The overall flow of the diagnostic framework.

The deep transfer learning method proposed in this study to address the S&I-IFD problem is illustrated in Figure 2. Unlike traditional fault diagnosis methods, this approach uses industrial data as the target domain and laboratory data as the source domain to aid in training intelligent diagnostic models. This method overcomes the limitations of small and imbalanced data in the target domain, enabling more effective handling of the S&I-IFD problem.

The whole S&I-IFD process can be divided into four steps:

Step 1: Data Segmentation. The time-series data from both the source and target domains are divided into samples of equal length using the sliding window method. To address the class imbalance in the target domain, different sliding window step sizes are applied to balance the number of samples for each class.

Step 2: Network Pre-training. The source domain data are split into training and validation sets. The ResFCN deep network model is then trained using the source domain training set and validated with the source domain validation set to ensure the accuracy of the pre-trained model.

Step 3: Model Transfer. Based on the transfer strategy, the pre-trained model is fine-tuned using the target domain training data to adapt it for S&I-IFD tasks.

Step 4: Model Testing. The trained S&I-IFD model is evaluated by classifying the target domain test data.

### 3.3. Data Segmentation

**Figure 3:**
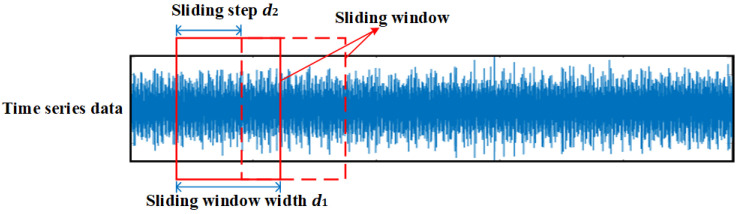
Schematic diagram of sliding window-based data segmentation.

Sensor data collected in industrial environments are typically time-series data. Before applying neural networks for deep feature extraction, data segmentation methods are often employed to divide the data into time segments of a specific length. It is important to note that any missing data or anomalies should be addressed through appropriate data cleaning before this step. One commonly used method for segmentation is the sliding window (SW), which has been widely applied in pattern recognition and other tasks. The two key parameters in SW are the sliding window width $d_{1}$ and the sliding step $d_{2}$. As shown in Figure 3, the sliding window width represents the segmentation length, while the sliding step indicates the length of each movement of the window during data reading.

In this study, the sliding window width d₁ is set to 100, which corresponds to 0.01 s based on the sampling rate of 10 kHz. This value is chosen to ensure that sufficient temporal information is captured for feature extraction. The sliding step d_2_ is determined to balance the trade-off between computational efficiency and the number of samples.

Therefore, for a time series of length l, segmented by a window width of $d_{1}$ and step of $d_{2}$, the number of samples n can be obtained as shown in Equation (3), where [ . ] is the rounding function.

(3) $$n=\left[\begin{array}{c} \frac{l-d_{1}}{d_{2}}+1 \end{array}\right]$$

For a fixed time series, the narrower the width of the window and the smaller the step size, the greater the number of samples obtained by segmentation, so the number of samples can be adjusted by adjusting these two parameters. Since the data in the target domain present an imbalanced state, the window width is kept constant and different step lengths are chosen for different classes of data so as to balance the number of samples from different classes and facilitate the feature extraction of the subsequent deep network. Since the data in the target domain present an imbalanced state, the window width is kept constant at 100 (0.01 s), and different step lengths are chosen for different classes of data to balance the number of samples from different classes and facilitate the feature extraction of the subsequent deep network.

### 3.4. Network Pre-Training

The method proposed in this study uses the residual FCN (ResFCN) model to address the S&I-IFD problem. FCNs are known for their robustness in handling time-series data, and the deep ResFCN network developed in the study presented herein excels in feature extraction and transferability, providing a strong foundation for subsequent transfer learning. In deep neural networks, the shallow layers typically extract broad, macroscopic features, while the deeper layers focus on more task-specific features. However, features extracted in the shallow layers can overwhelm deeper layers, reducing their ability to extract meaningful information and potentially hindering the model’s performance. To address this, we incorporate a residual module that facilitates better integration of shallow features into deeper layers, thus mitigating issues like gradient vanishing and explosion and slowing down overfitting.

**Figure 4:**
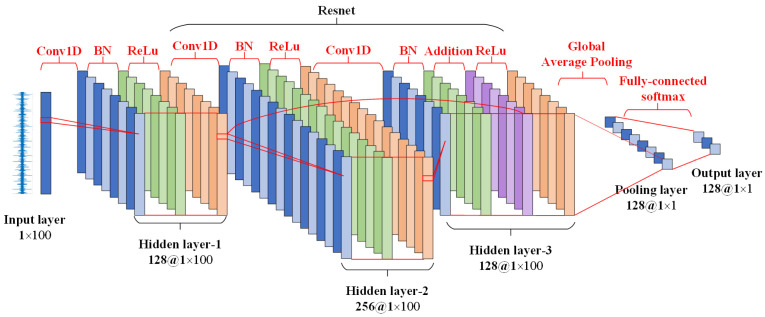
The schematic diagram of the residual fully convolutional network (ResFCN) structure used in the method proposed in this study. The network mainly consists of a convolutional layer, a batch normalization layer, an activation layer, a global average pooling layer, and a residual structure, which is good at extracting the potential features of the input signal.

A schematic diagram of the ResFCN structure is shown in Figure 4. The model takes a variable-length time series as input and outputs a class prediction for the data. The input layer is followed by three hidden layers, each performing a series of three operations: convolution, batch normalization, and activation.

The method proposed in this study leverages the residual FCN (ResFCN) model to address the S&I-IFD problem. FCNs are known for their robustness in time-series tasks, and the deep ResFCN network developed in the study presented herein offers strong feature extraction capabilities and high transferability, which enhances subsequent transfer learning. In deep neural networks, shallow layers typically extract broad, macroscopic features, while deeper layers focus on more specific features. However, the shallow-layer features can sometimes overwhelm the deeper layers, as shown in Equation (4), reducing their impact and impairing the model’s ability to extract useful information. To address this, we introduce a residual module that effectively integrates shallow features into the deeper layers, mitigating issues like gradient vanishing and explosion and slowing down overfitting.

(4) $$\mu_{B}\leftarrow \frac{1}{m}\sum_{i=1}^{m}x_{i}$$

Subsequently, the variance of each training batch data is found according to Equation (5).

(5) $$\sigma_{B}^{2}\leftarrow \frac{1}{m}\sum_{i=1}^{m}{\left(x_{i}-\mu_{B}\right)}^{2}$$

Then, the training data of the batch are normalized according to Equation (6) using the obtained mean and variance to obtain a (0,1) normal distribution, where ϵ is the tiny positive number used to avoid the divisor being zero.

(6) $${\hat{x}}_{i}\leftarrow \frac{x_{i}-\mu_{B}}{\sqrt{\sigma_{B}^{2}+\epsilon }}$$

Finally, scale transformation and offset are performed according to Equation (7) so that the normalized $x_{i}$ is restricted to be under the normal distribution.

(7) $$y_{i}\leftarrow \gamma {\hat{x}}_{l}+\beta ={\mathrm{BN}}_{\gamma ,\beta }\left(x_{i}\right)$$

The specific hyperparameters of the three convolutional layers are shown in Table 1. This is followed by a pooling layer, which performs a global averaging operation, that is, the results of the previous layer are averaged over the time axis. The pooling layer combines the statistical feature values of multiple pixels in the pooled region instead of using the value of each pixel, which makes the pooling unit less than the detection unit and thus reduces the computational burden. Also, since the next layer of the pooling layer is a fully connected layer, this reduces the input size, thus serving to improve statistical efficiency and reduce the storage requirements for parameters. The output layer uses SoftMax to perform the classification and the number of neurons in this layer is equal to the number of classes C. Also, the residual structure is used in hidden layer 1 and hidden layer 3 to prevent gradient disappearance, gradient explosion, and overfitting while better preserving the shallow features. Thus, the output of hidden layer 3 $hidden_{3}$ is shown in Equation (8), where $hidden_{1}$ denotes the output of hidden layer 1 and $BN_{3}$ denotes the output of the BN layer in hidden layer 3.

(8) $$hidden_{3}=hidden_{1}+BN_{3}$$

In the network pre-training phase, the source domain dataset is first divided into a training set and a validation set. A smaller number of epochs is initially selected, and the network is trained using the training set. The validation set is then input into the trained model for evaluation. If the accuracy meets the required threshold, the pre-training process is complete. Otherwise, the model is retrained using the training set until the desired accuracy is achieved. By choosing a small number of epochs, overfitting is avoided during the training process. This gradual training ensures that the pre-trained network reaches an optimal state, neither underfitting nor overfitting, thereby improving generalization and enabling the accurate extraction of fault-related features from the source domain.

The proposed method involves multiple preprocessing steps and transfer learning processes, which may increase computational overhead. To address this issue, the computational complexity of the ResFCN model and the associated preprocessing steps is analyzed. Preprocessing mainly includes standardizing the time-series data and preparing the source and target datasets for transfer learning. These steps have a complexity that is linear with respect to the size of the dataset, $O\left(n\right)$, where n is the number of data points in the time series. The computational complexity of the ResFCN model depends on the number of convolutional layers, the number of filters, and the length of the input time series. For each convolutional layer, the complexity is approximately O(k⋅m⋅f), where k is the filter length, m is the input sequence length, and f is the number of filters. Based on the hyperparameters in Table 1, the complexities of the three convolutional layers are O(8⋅m⋅128), O(5⋅m⋅256), and O(3⋅m⋅128). In addition, the global average pooling layer has a complexity of $O\left(m\right)$, as it only averages the values along the time axis, while the Softmax layer has a complexity of $O\left(c\right)$, where c is the number of classes. In summary, the overall complexity of the ResFCN model is primarily determined by the convolutional layers, with a complexity of O(k⋅m⋅f). The use of residual structures and the global average pooling layer reduces the computational burden to some extent while improving the training efficiency and feature extraction capability of the model. Furthermore, the pretraining process in transfer learning adopts a small number of epochs, which further reduces the computational overhead, ensuring that the model achieves high performance while maintaining manageable computational complexity.

### 3.5. Transfer Strategy

Since the distribution of the source and target domain data differ significantly, the pre-trained model cannot be directly applied to the intelligent diagnosis of the target domain data. To address this, this paper uses a transfer learning approach to fine-tune the pre-trained model with a small amount of target domain data, extracting cross-domain invariants and ultimately achieving intelligent fault diagnosis

**Figure 5:**
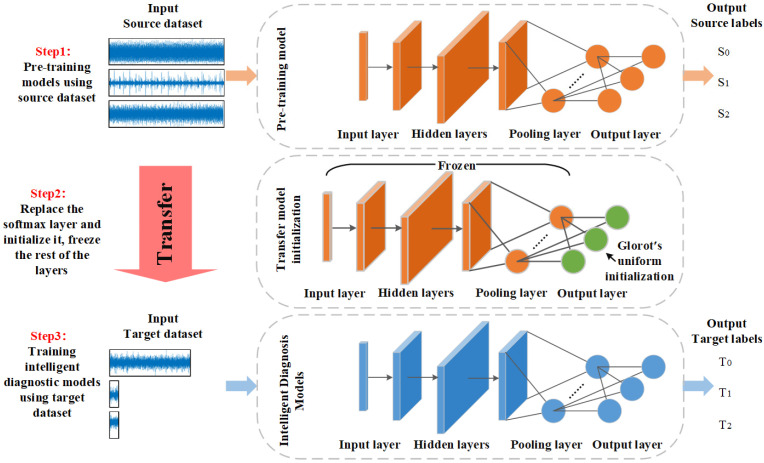
Schematic diagram of the transfer strategy used in the study. The transfer strategy is divided into three steps: step 1 is to pre-train using the source domain data to obtain the pre-trained model; step 2 is to replace the softmax layer of the pre-trained model, initialize it, and freeze all other layers; and step 3 is to train using the target domain data to obtain the intelligent diagnostic model.

The schematic diagram of transfer learning is shown in Figure 5. First, the pre-training model is obtained by training with the source domain data. Then, the softmax layer of the pre-trained neural network is removed and replaced with another softmax layer with the number of neurons equal to the number of classes in the target domain. The added softmax layer is randomly initialized using Glorot’s uniform initialization method. Glorot’s uniform distributes the data uniformly over the interval [−limit,limit], with limit taking the value shown in Equation (9). Using this model as a base, it is retrained on $D^{t}$.

(9) $$limit=sqrt\left(\frac{6}{fan_{in}+fan_{out}}\right)$$

where $fan_{in}$ represents the number of input neurons and $fan_{out}$ represents the number of output neurons.

To improve learning and prevent the model from stalling, a callback function is employed to monitor the loss changes during training. When learning stagnates, reducing the learning rate by a factor of 2–10 can help the model tune more accurately and improve performance. We set the change in learning rate to be triggered when $n_{lr}$ epochs pass without any improvement in model performance. As shown in Equation (10), at each change, the learning rate becomes f times the previous one, with a lower bound of $lr_{min}$.

(10) lr=lr∗f

The quantity used to measure the model performance is the cross-entropy loss, which portrays the distance between two probability distributions. Let there be $n_{s}$ labeled samples and C classes, and the output of the neural network corresponding to each sample $x_{i}$ is $\left({\hat{y}}_{i,1},{\hat{y}}_{i,2},\ldots ,{\hat{y}}_{i,C}\right)$, which represents the probability that $x_{i}$ belongs to each class in the predicted outcome. Then, the formula of cross entropy loss is shown in Equation (11), where $y_{i,j}$ is a symbolic function, and the true class of sample $x_{i}$ is taken as 1 if it is equal to j, otherwise it is taken as 0.

(11) $$L=\frac{1}{N}\sum_{i}\sum_{j=1}^{C}y_{i,j}\log({\hat{y}}_{i,j})$$

To ensure the model’s convergence, in this study, we adjust the parameters of the entire network rather than just those of the final softmax layer. This approach leads to a more significant improvement in diagnostic accuracy for the target domain compared to simply initializing all the weights.

## 4. Experiments

### 4.1. Dataset and S&I-IFD Scenario Description

In this study, we used bearing data to demonstrate the usability and accuracy of the proposed S&I-IFD method. The source domain data, representing laboratory data, and the target domain data, representing real equipment data, are derived from two different publicly available bearing datasets that are widely used in research in this field, ensuring the authenticity and accuracy of the data. The details are as follows:

The source domain data were obtained from the Bearing Data Center at Case Western Reserve University (CWRU) [37]. These data consist of acceleration signals measured at the drive-side and fan-side bearing positions under four different motor loads, with sampling frequencies of 12 kHz and 48 kHz. For this study, three types of data (normal condition, inner race fault, and outer race fault) with a sampling frequency of 48 kHz were selected, and the appropriate data length was chosen for the experiment. The specific parameters are shown in Table 2.

The target domain data were obtained from the Paderborn University (PU) Bearing Data Center [38]. These data were collected from the vibration signal of the bearing seat using a piezoelectric accelerometer with a sampling frequency of 64 kHz. In this study, three types of data (normal condition, inner race fault, and outer race fault) were selected. The training data consist of 10 times the normal data compared to the fault data, and the test data are set to the same length. The specific parameters are shown in Table 3.

**Figure 6:**
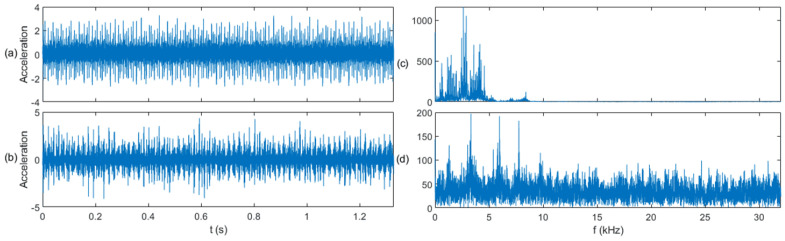
Signal comparison of the normal class: (**a**) time domain waveform in the source domain, (**b**) time domain waveform in the target domain, (**c**) FFT spectrum in the source domain, and (**d**) FFT spectrum in the target domain.

**Figure 7:**
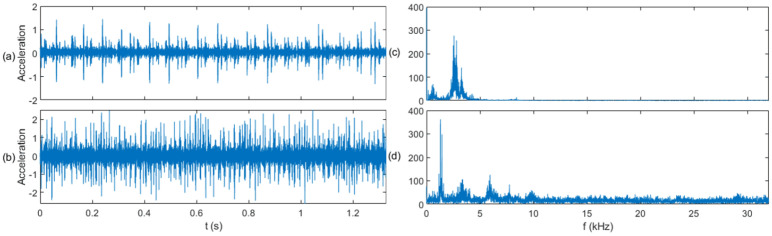
Signal comparison of the inner race fault class: (**a**) time domain waveform in the source domain, (**b**) time domain waveform in the target domain, (**c**) FFT spectrum in the source domain, and (**d**) FFT spectrum in the target domain.

**Figure 8:**
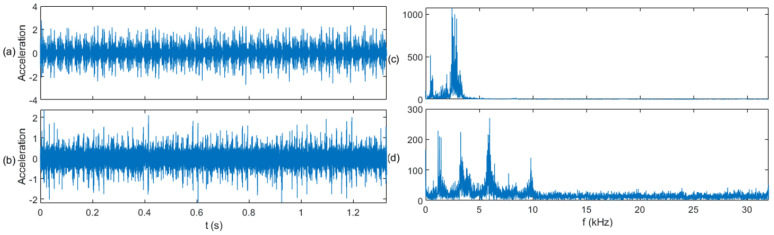
Signal comparison of the outer race fault class: (**a**) time domain waveform in the source domain, (**b**) time domain waveform in the target domain, (**c**) FFT spectrum in the source domain, and (**d**) FFT spectrum in the target domain.

In the S&I-IFD experiment, we constructed a model based on the CWRU dataset (source domain) and transferred it to the PU dataset (target domain). The time waveforms and fast Fourier transform (FFT) spectra of the three types of signals in both the source and target domains are shown in Figure 6, Figure 7 and Figure 8. From a fault classification perspective, distinguishing between normal, inner race fault, and outer race fault is challenging, particularly because the FFT spectra of inner and outer race faults are very similar, making fault diagnosis difficult. From the standpoint of data similarity between the source and target domains, the data distributions in both the time and frequency domains are significantly different. It is challenging to find correlations between the source and target domains when comparing them intuitively in these domains, which makes transfer learning difficult. This experiment will demonstrate that the proposed diagnostic method can effectively leverage fault-related information across the two domains.

The application of the sliding window (SW) method significantly enhances the correlation between the source and target domains by segmenting the time-series data into uniform samples. This segmentation not only balances the data distribution in the target domain but also highlights shared temporal and spectral features between the two domains. For example, as shown in Figure 6, Figure 7 and Figure 8, SW-based segmentation reduces the overlap between the inner and outer race fault spectra, making it easier to distinguish between fault types. This improvement in feature separability is critical for the subsequent transfer learning process, as it ensures that the pre-trained model can effectively adapt to the target domain data.

### 4.2. S&I-IFD Experimental Procedure and Results

In this section, the usability and accuracy of the S&I-IFD method proposed in this paper are investigated using source and target domain data.

#### 4.2.1. Experimental Procedure

Using the method proposed in Section 3, S&I-IFD model training is completed using the source domain data aided by the target domain training data. The specific steps and related parameters are as follows.

Step 1: Data Segmentation. The data for each class in both the source and target domains are partitioned into equal-length samples using the sliding window (SW) method. The sliding window width is set to 100, and the sliding step and the number of samples after segmentation are shown in Table 4. The table presents the data in the following order: (normal condition, inner race fault, and outer race fault).

Step 2: Network Pre-training. The source domain samples are split into a training set and a validation set with a 9:1 ratio. The training set is used to train the ResFCN model for a specified number of epochs, resulting in a pre-trained model. The validation set is then used to verify the model’s performance. If the model’s accuracy on the validation set exceeds the predefined critical accuracy threshold, training stops; otherwise, training continues. The hyperparameters used during training are listed in Table 5.

Step 3: Model Transfer. The softmax layer of the pre-trained model is removed and replaced with a new one, initialized randomly using Glorot’s uniform initialization method. The target domain training samples are then fed into the initialized model, and transfer learning is applied according to the transfer strategy to obtain the final S&I-IFD model. To enhance learning, a callback function monitors loss changes, and when 50 epochs pass without improvement in model performance, the learning rate is halved, with a lower bound of 0.001.

Step 4: Model Testing. The trained S&I-IFD model is tested by inputting the target domain test samples. The predicted values are compared with the actual values to calculate the model’s accuracy and generate the confusion matrix.

Experimental Setup. The experiments were developed in Python. The equipment used consists of a PC with an Intel Core i7-8565U CPU (1.80 GHz), 4 GB of RAM, and an NVIDIA GeForce MX230 graphics card.

#### 4.2.2. Results and Analysis

**Figure 9:**
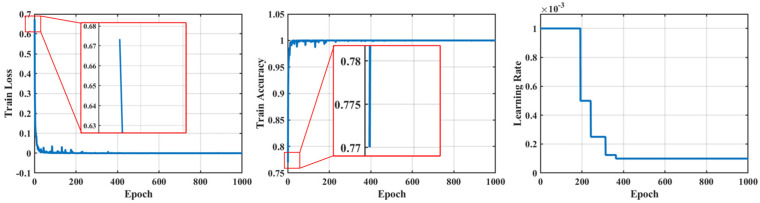
The curves of training set loss, training set accuracy, and learning rate with the number of training sessions during transfer learning.

The curves of training loss, training accuracy, and learning rate during transfer learning are shown in Figure 9. At the beginning of the transfer learning process, the model’s accuracy on the target domain is low, and the loss is high due to the significant difference between the source and target domain data. As training progresses, the accuracy on the training set improves and stabilizes around 1 after approximately 500 epochs, indicating that the model has become well-suited for fault diagnosis in the target domain. As training continues, the learning rate is reduced by half whenever the model’s performance stagnates, facilitating further fine-tuning.

This study visualizes the diagnostic results using a confusion matrix. In the confusion matrix chart, rows correspond to predicted classification, columns correspond to true classification, diagonal lines correspond to observations for correct classification, and off-diagonal lines correspond to observations for incorrect classification, and percentages of the number of observations and a total number of observations are shown in each cell. The rightmost column of the chart shows the percentages of all examples for all categories for both correct and incorrect classifications. The bottom row of the chart shows the percentage of all examples belonging to each category that were correctly and incorrectly categorized. The cell at the bottom right of the graph shows the overall accuracy.

We define that for each class of sample, the following four classification cases exist: positive samples predicted by the model as positive (True Positive), negative samples predicted by the model as positive (False Positive), positive samples predicted by the model as negative (False Negative), and negative samples predicted by the model as negative (True Negative). Therefore, we use the accuracy, precision, recall, and F1-score as the evaluation metrics of the classification results. As shown in Equation (12), the accuracy reflects the classifier’s ability to determine the entire sample. As shown in Equations (13)–(15), the precision indicates the proportion of true positive samples among the positive examples determined by the classifier, the recall indicates the proportion of correctly determined positive examples to the total positive examples, and the F1-score is a summed average of the precision and recall. The performance of the classifier on the entire dataset is also evaluated by macro-average, as shown in Equations (16)–(18), which is a weighted average of the precision, recall, and F1-score for each degradation state.

To ensure the generalizability and reliability of the experimental results, the experiments were repeated 10 times and the model was evaluated using the average of the 10 experiments.

(12) $$A=\frac{Number\;of\;correctly\;predicted\;samples}{Total\;number\;of\;samples}$$

(13) $$P_{i}=\frac{TP_{i}}{TP_{i}+FP_{i}}$$

(14) $$R_{i}=\frac{TP_{i}}{TP_{i}+FN_{i}}$$

(15) $$F1_{i}=\frac{2P_{i}R_{i}}{P_{i}+R_{i}}$$

(16) $$P_{\mathrm{Macro}\;\mathrm{avg}\;}=\frac{1}{C}\sum_{i}P_{i}$$

(17) $$R_{\mathrm{Macro}\;\mathrm{avg}\;}=\frac{1}{C}\sum_{i}R_{i}$$

(18) $$F1_{\mathrm{Macro}\;\mathrm{avg}\;}=\frac{1}{C}\sum_{i}F1_{i}$$

**Figure 10:**
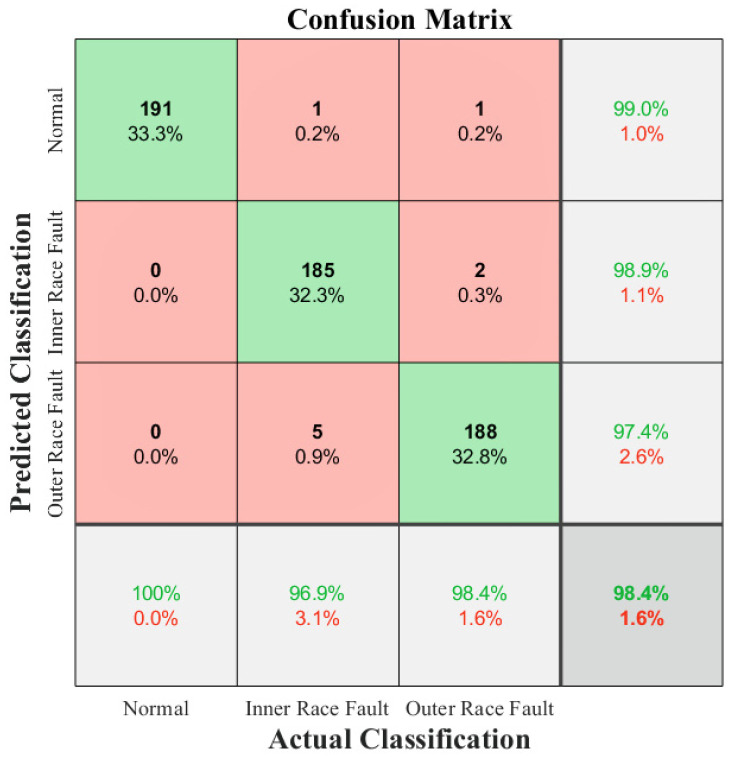
The confusion matrix of the experimental results.

The confusion matrix of the experimental results is shown in Figure 10, while the accuracy, precision, recall, and F1-score are presented in Table 6. We can see that the method proposed in this study uses source domain data to assist in training with small and imbalanced training data in the target domain, and the final classification accuracy of the model obtained is 98.43%. In the classification of each state, all the data of the normal condition are correctly classified, and 96.86% and 98.43% of the data of each of the two fault states are correctly classified, and the classification accuracy of each state is above 97%. This indicates that the method proposed in this study can use a large amount of balanced source domain data to assist training in the case of small and imbalanced training data in the target domain, using a reasonable network model and an effective transfer learning method, and has good feasibility and high accuracy in the research framework proposed in this study.

### 4.3. Comparison of the Different Steps of the S&I-IFD Method

#### 4.3.1. Experimental Procedure

In order to demonstrate the necessity of each step in the method proposed in this study, the results of different experiments are compared in this section by removing specific steps. There are three main steps in the method proposed in this study: data segmentation of time-series data using SW; model pre-training using source domain data; and fine-tuning of the pre-trained model using transfer learning. We removed one or two of these steps, and together with the method proposed in this study, there are six tasks, as shown in Table 7.

Experiments for tasks 1–6 were conducted using the same dataset and within the same computational environment. For each task, this study evaluates performance based on 13 indicators: 9 indicators related to the classification performance of each class (precision, recall, and F1-score for each of the normal condition, inner race fault, and outer race fault) and 4 overall evaluation metrics (macro-average precision, recall, and F1-score, as well as overall accuracy). To ensure the robustness of the results, 10 independent experiments were conducted for each task, and the average performance across these 10 experiments is reported.

#### 4.3.2. Results and Analysis

**Figure 11:**
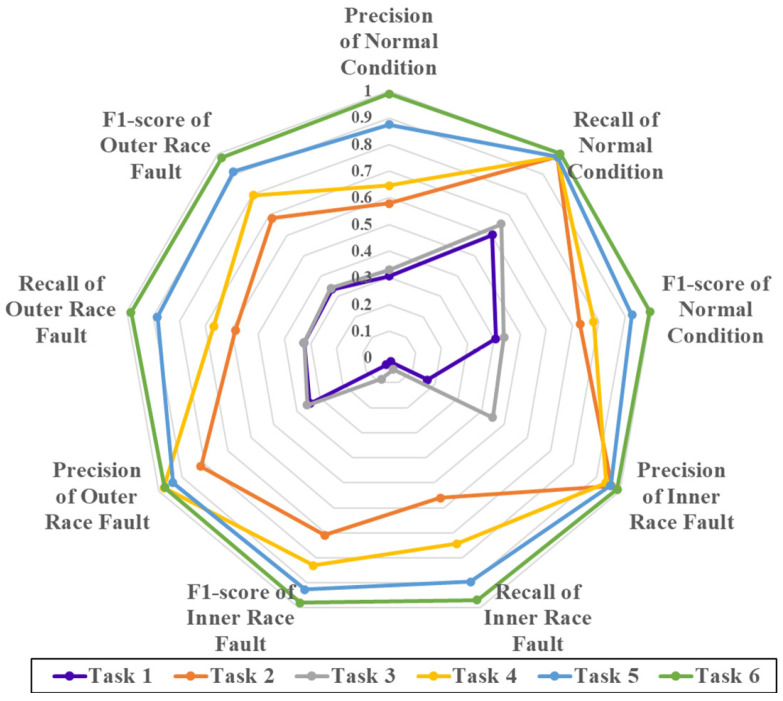
Results of the 6 tasks on 9 indicators describing the classification of each category. On each indicator, the closer the point is to the center, the lower the accuracy.

**Figure 12:**
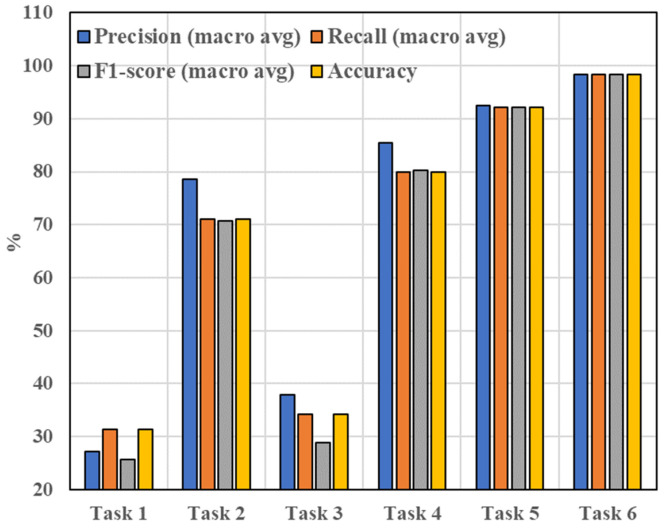
Results of 6 tasks on 4 macro-averages (precision, recall, F1-score, and accuracy) (in percentage).

The results of the experiments are shown in Figure 11 and Figure 12. Tasks 1 and 3 both involve training using only source domain data, and the resulting models were directly tested on the target domain data. Both tasks showed poor performance across all evaluation metrics. Specifically, when examining the accuracy, recall, and F1-score for each class, both models classified almost all data as belonging to the “normal” class, indicating that the classifiers are not suitable for the target domain. The overall accuracy for both methods was approximately 32%, demonstrating that the models hardly serve any useful classification purpose. This poor performance is attributed to the significant difference in data distribution between the source and target domains. A model trained solely on source domain data can only extract features relevant to the source domain and is unable to generalize effectively to the target domain. While both domains contain relevant fault-related features, training only on the source domain without fine-tuning on the target domain data results in models that cannot accurately diagnose faults in the target domain, highlighting the need for transfer learning. Additionally, Task 3 uses sliding window (SW) for data segmentation more than Task 1, leading to a slight improvement in accuracy, though the difference is not significant.

Tasks 5 and 6 both involve training the model with source domain data and fine-tuning it using target domain training data, followed by testing on target domain test data. These methods show much better performance, with accuracy, recall, and F1-scores for each class indicating that the models can classify the majority of the data correctly. Both tasks achieve accuracy rates exceeding 90%, demonstrating their effectiveness in performing fault diagnosis. This improved performance can be attributed to the combination of large, balanced source domain data and smaller target domain data. The use of model pre-training allows the network to capture fault-related information from the source domain, while transfer learning enables the model to adapt and find common features across the two domains, compensating for the small and imbalanced target domain data. This process also avoids the negative transfer effects caused by the differences in data distribution between the source and target domains. Task 6, which uses more SW for data segmentation than Task 5, results in an accuracy increase from 92% to 98%, further demonstrating that SW helps mitigate the impact of data imbalance and positively influences the final model’s performance.

Overall, the results from these six tasks clearly demonstrate the necessity of the three main steps in the proposed method—data segmentation using SW, model pre-training with source domain data, and fine-tuning with transfer learning. Each step plays a crucial role in improving the model’s effectiveness and accuracy in fault diagnosis.

### 4.4. Comparison with Other Relevant Research Methods

#### 4.4.1. Experimental Procedure

In order to demonstrate the superiority of the proposed method in this study, we compared it with nine classical underlying models in related fields. Detailed descriptions of these methods and special parameter settings are as follows.

1. The CNN consists of a feature extractor and a label classifier and is a classical neural network. Optimization is performed using random search for relevant hyperparameters.
2. Long short-term memory network (LSTM) is a commonly used method in time-series data to avoid the long-term dependency problem.
3. ResFCN is the network model used in this study, and the experimental results using this model are used to compare whether transfer learning will result in negative transfer cases.
4. Deep neural networks (DNNs) are a commonly used neural network in deep learning, which are optimized using stochastic search for relevant hyperparameters.
5. TCA [39] is a classical method in transfer learning, which adaptively draws the probability distribution of the data in the source and target domains through edge distribution, so as to achieve the transfer of the model. Here, the RBF kernel is chosen to calculate the maximum mean difference (MMD).
6. Balanced distribution adaptation (BDA) [40] can consider both edge distribution and conditional distribution and adjust the importance of both adaptively according to the data characteristics in the source and target domains. Here, the RBF kernel is chosen to calculate the MMD.
7. Geodesic flow kernel (GFK) cite [41] is a streaming learning method that can efficiently implement transfer between two domains.
8. Correlation alignment (CORAL) [42] is a statistical feature alignment method that aligns the source and target domains with second-order features, thus reducing the differences in distribution.
9. The domain adaptive neural network (DANN) [43] consists of a feature layer and a classifier layer and adds an MMD adaptation layer after the feature layer for computing the distance between the source and target domains.

Four of them are methods without transfer learning (CNN, LSTM, ResFCN, and DNN) and five are transfer learning methods (TCA, BDA, GFK, CORAL, DANN). For CNN, LSTM, ResFCN, and DNN, no transfer learning is performed and the models are trained directly using the target domain training data, and the trained models are tested using the target domain test data. For TCA, BDA, GFK, CORAL, and DANN, the basic network structure used is ResFCN, and the same source and target domain data are used as before. The models are trained using the source domain data, subsequently transferred to the target domain, and finally tested using the target domain test data.

For each method, this study evaluates 13 indicators. There are 9 indicators used to describe the classification of each class (precision, recall, and F1-score corresponding to each of the normal condition, inner race fault, and outer race fault), and 4 overall evaluation indicators (macro-average of precision, recall, and F1-score, and accuracy). To ensure the generalizability of the results, 10 experiments were performed for each method, and the 10 results were averaged.

#### 4.4.2. Results and Analysis

**Figure 13:**
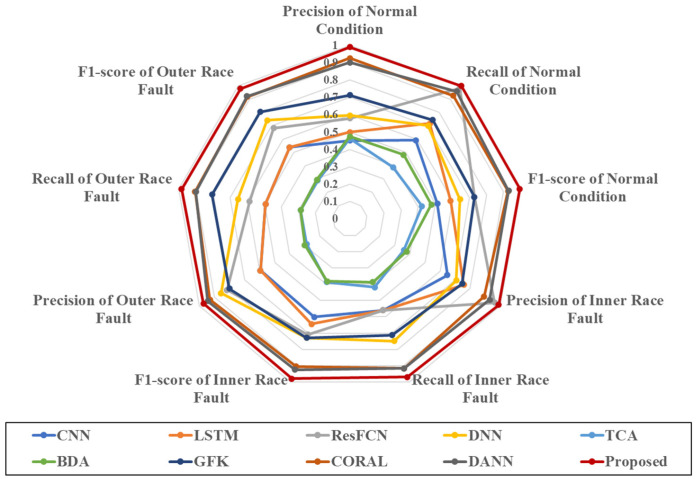
Results of the 10 methods on 9 indicators describing the classification of each category. On each indicator, the closer the point is to the center, the lower the accuracy.

**Figure 14:**
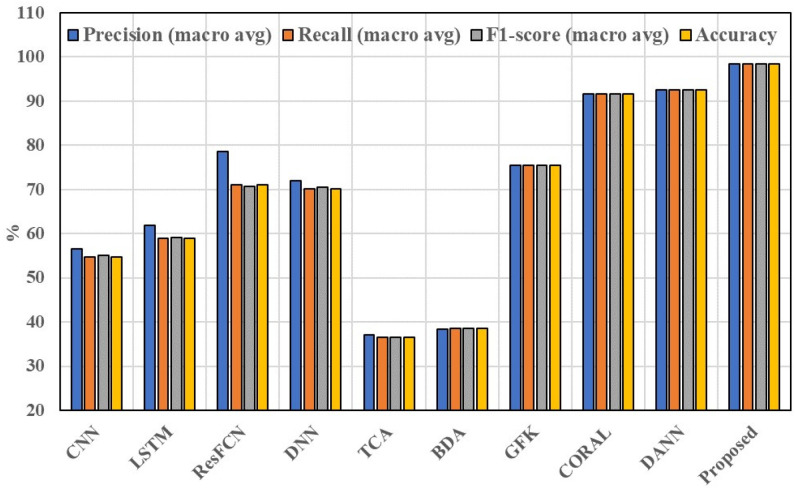
Results of 10 methods on 4 macro-averages (precision, recall, F1-score, and accuracy) (in percentage).

Figure 13 and Figure 14 plot the overall comparison results of the 10 methods. As basic classifiers, the results of CNN and LSTM are similar without using transfer learning, and the accuracy of the models is 54.8% and 58.99%, respectively, with relatively low classification accuracy. ResFCN and DNN have relatively better results, with the accuracy of the models being around 71%, and the results are similar in the classification of each category. All four methods do not use source domain data but rather target domain data for training and testing. Affected by the small and imbalanced training data in the target domain, the classification effect of the model is a great limitation and the accuracy is not high.

### 4.5. Other Transfer Scenarios

#### 4.5.1. Experimental Procedure

To better demonstrate the generalization and scalability of the S&I-IFD method proposed in this study, 16 different transfer scenarios were designed based on two datasets, CWRU and PU. The source data were obtained from the CWRU dataset, as shown in Table 8 for the CWRU dataset selected for this study, and they have different working conditions and sensor locations. CWRU-DE-L and CWRU-DE-H are collected by sensors at the drive end (DE) of the bearing, and CWRU-FE-L and CWRU-FE-H are collected by sensors at the fan end (FE) of the bearing. L and H are used to indicate the operating conditions of the bearings, CWRU-DE-L and CWRU-FE-L are collected at a load of 0 kW and a rotation speed of 1797 r/min, and CWRU-DE-H and CWRU-FE-H are collected at a load of 2.21 kW and a rotation speed of 1730 r/min. The target data are obtained from the PU dataset, as shown in Table 9, and their working conditions and damage setting modes are different. The damage setting mode for PU-EE-L and PU-EE-H is manual electric engraver (EE), and the damage setting mode for PU-EDM-L and PU-EDM-H is electrical discharge machining (EDM). Similarly, L and H are used to indicate the operating conditions of the bearing. PU-EE-L and PU-EDM-L are taken at a torque of 0.1 Nm and a rotation speed of 1500 r/min, while PU-EE-H and PU-EDM-H are taken at a torque of 0.7 Nm and a rotation speed of 1500 r/min.

Therefore, 16 transfer learning tasks with different transfer scenarios were constructed, as shown in Table 10. The source domain data of these tasks are all from the CWRU dataset and the target domain data are all from the PU dataset, and they all need to complete the fault diagnosis across working conditions and machines. The number of data samples and the selection of the number of sample points in each sample, the setting of hyperparameters, and the selection of evaluation indicators are the same as in Section 4.2.

#### 4.5.2. Results and Analysis

The results of the proposed method in this study on 16 transfer tasks are shown in the table. As can be seen from Table 11, the final accuracies of all 16 tasks exceed 97% with an average accuracy of 98.43% under different transfer scenarios, despite the differences in the working conditions, sensor locations, and fault setting methods in the source and target domain data. From the accuracy, recall, and F1-score of each category, the method proposed in this study achieves relatively stable classification results in all 16 tasks, and the average value of each index is above 98%, which is already a relatively good level for a small sample fault diagnosis scenario across working conditions and machines, and the data imbalance condition reaches 10:1. This indicates that the method proposed in this study can sufficiently explore the potentially effective information contained in the small sample data to achieve accurate fault diagnosis, thus proving the usability, accuracy, and stability of the method in the bearing cross-machine fault diagnosis scenario.

In comparison, existing methods face significant challenges in addressing cross-machine small-sample and imbalanced data fault diagnosis. Traditional transfer learning methods often suffer from negative transfer due to the large discrepancies in operating conditions and structures between the source and target domains. Additionally, the severe imbalance in target domain data, where normal condition data far outnumber fault data, makes it difficult to effectively extract fault-related features, leading to biased predictions toward the majority class. The proposed method overcomes these limitations by leveraging limited target domain data and extracting meaningful cross-domain features. Through the integration of sliding window segmentation and the ResFCN model, the method achieves robust and accurate fault diagnosis in complex cross-machine scenarios, demonstrating strong adaptability and reliability.

## 5. Conclusions

In this paper, we propose a new deep transfer learning method for equipment fault diagnosis. Since it is difficult to build an effective intelligent diagnosis model due to the small and imbalanced samples in real industrial scenarios, we used laboratory data with large and balanced data to assist in training. The target domain data were initially balanced using the SW-based data segmentation method. The universal features associated with faults in the source domain data are effectively mined by the ResFCN network. The pre-trained model is fine-tuned using transfer learning to learn personalized features that better match the target domain. Experimental results using two publicly available datasets show that the method can better span the data differences between the source and target domains in terms of equipment working conditions, sizes, and models and achieve effective feature extraction and high diagnostic accuracy. Compared with other popular advanced methods, this method has obvious advantages.

The limitation of this study is that the experiments used to validate the proposed method involved the use of publicly available datasets without building equipment and acquiring data in a personal laboratory. In the future, we will build complex and representative bearing equipment, acquire data, and conduct relevant theoretical studies.

## Figures and Tables

**Table 1:** Specific hyperparameters of the three convolutional layers.

Hyperparameter	Convolutional Layer 1	Convolutional Layer 2	Convolutional Layer 3
Number of filters	128	256	128
Filter length	8	5	3
Stride		1	
Padding	Preserve the convolution result at the boundary		

**Table 2:** Parameters related to source domain data.

Rotation Speed (r/min)	Load (kw)	Fault	Fault Diameter (mm)	Data Volume
1797	0	Normal condition	\	62,600
		Inner race fault	0.1778	21,200
			0.3556	21,200
			0.5334	21,200
		Outer race fault	0.1778	21,200
			0.3556	21,200
			0.5334	21,200

**Table 3:** Parameters related to target domain data.

Rotation Speed (r/min)	Load (kw)	Fault	Fault Diameter (mm)	Data Volume	Test Data Volume
1500–2000	\	1000–3000	Normal condition	44,500	19,100
1500	0.1	1000	Inner race fault	4400	19,100
	0.1	1000	Outer race fault	4400	19,100

**Table 4:** The values of the sliding step $d_{2}$ and the number of samples $n$ after segmentation in the form of (normal condition, inner race fault, and outer race fault).

	Source Domain	Target Domain Train Dataset	Target Domain Test Dataset
Sliding step	(100, 100, 100)	(100, 9, 9)	(100, 100, 100)
Number of samples	(626, 626, 626)	(445, 478, 478)	(191, 191, 191)

**Table 5:** Hyperparameter settings during the training process.

Hyperparameter	Value	Hyperparameter	Value
Epochs	(100, 100, 100)	Learning rate	0.001
Batch size	16	Loss function	Cross-entropy
Optimizer	Adam	Critical accuracy	0.99

**Table 6:** The results of accuracy, precision, recall, and F1-score in the experiments.

	Precision (%)	Recall (%)	F1-Score (%)
Normal condition	98.96	100.00	99.48
Inner Race Fault	98.93	96.86	97.88
Outer Race Fault	97.41	98.43	97.92
Macro avg	98.43	98.43	98.43
Accuracy (%)	98.43		

**Table 7:** The six tasks resulting from the removal of one or some steps used to examine the necessity of each step of the method proposed in this paper.

Hyperparameter	Value
Task 1	**Without using SW,** the original data are directly partitioned into equal-length samples. **Train with source domain data** and test the trained model directly with the target domain test data.
Task 2	**Without using SW,** the original data are directly partitioned into equal-length samples. **Train with the target domain training data** and test the completed model directly using the target domain test data.
Task 3	**Use SW for data segmentation. Train with source domain data** and test the completed model directly with the target domain test data.
Task 4	**Use SW for data segmentation. Train with the target domain training data** and test the completed model directly with the target domain test data.
Task 5	**Without using SW,** the original data are directly partitioned into equal-length samples. **Train with the source domain data and fine-tune with the target domain training data,** and test the fine-tuned model with the target domain test data.
Task 6	**Using SW,** the original data are directly partitioned into equal-length samples. **The model is trained with the source domain data and fine-tuned with the target domain training data,** and the fine-tuned model is tested with the target domain test data.

**Table 8:** Introduction of source domain data from the CWRU dataset.

Datasets	Load (kW)	Rotation Speed (r/min)	Sensor Position
CWRU-DE-L	0	1797	DE
CWRU-DE-H	2.21	1730	DE
CWRU-FE-L	0	1797	FE
CWRU-FE-H	2.21	1730	FE

**Table 9:** Introduction of target domain data from the PU dataset.

Datasets	Torque (Nm)	Rotation Speed (r/min)	Failure Mode
PU-EE-L	0.1	1500	EE
PU-EE-H	0.7	1500	EE
PU-EDM-L	0.1	1500	EDM
PU-EDM-H	0.7	1500	EDM

**Table 10:** 16 transfer tasks.

Task	Source Data	Target Data	Task	Source Data	Target Data
T1	CWRU-DE-L	PU-EE-L	T9	CWRU-DE-L	PU-EDM-L
T2	CWRU-DE-H	PU-EE-L	T10	CWRU-DE-H	PU-EDM-L
T3	CWRU-FE-L	PU-EE-L	T11	CWRU-FE-L	PU-EDM-L
T4	CWRU-FE-H	PU-EE-L	T12	CWRU-FE-H	PU-EDM-L
T5	CWRU-DE-L	PU-EE-H	T13	CWRU-DE-L	PU-EDM-H
T6	CWRU- DE-H	PU-EE-H	T14	CWRU- DE-H	PU-EDM-H
T7	CWRU-FE-L	PU-EE-H	T15	CWRU-FE-L	PU-EDM-H
T8	CWRU-FE-H	PU-EE-H	T16	CWRU-FE-H	PU-EDM-H

**Table 11:** The results of 13 indicators for 16 tasks (in percentage).

Task	Normal Condition			Inner Ring Fault			Outer Ring Fault			Macro Avg			Accuracy
	Precision	Recall	F1-Score	Precision	Recall	F1-Score	Precision	Recall	F1-Score	Precision	Recall	F1-Score	
T1	8.96	100.00	99.48	98.93	96.86	97.88	97.41	98.43	97.92	98.43	98.43	98.43	98.43
T2	98.95	98.43	98.69	96.92	98.95	97.93	98.94	97.38	98.15	98.27	98.25	98.26	98.25
T3	99.48	99.48	99.48	98.95	98.95	98.95	98.95	98.95	98.95	99.13	99.13	99.13	99.13
T4	98.41	97.38	97.89	96.46	100.00	98.20	97.85	95.29	96.55	97.58	97.56	97.55	97.56
T5	98.39	95.81	97.08	97.94	99.48	98.70	96.89	97.91	97.40	97.74	97.73	97.73	97.73
T6	100.00	99.48	99.74	96.41	98.43	97.41	98.40	96.86	97.63	98.27	98.25	98.26	98.25
T7	98.92	95.81	97.34	98.95	98.43	98.69	96.46	100.00	98.20	98.11	98.08	98.08	98.08
T8	99.47	98.43	98.95	97.93	98.95	98.44	99.48	99.48	99.48	98.96	98.95	98.95	98.95
T9	99.47	97.38	98.41	99.47	98.43	98.95	96.95	100.00	98.45	98.63	98.60	98.60	98.60
T10	98.95	98.43	98.69	97.42	98.95	98.18	98.94	97.91	98.42	98.44	98.43	98.43	98.43
T11	97.91	97.91	97.91	97.89	97.38	97.64	99.48	100.00	99.74	98.43	98.43	98.43	98.43
T12	98.91	95.29	97.07	100.00	100.00	100.00	95.45	98.95	97.17	98.12	98.08	98.08	98.08
T13	99.48	99.48	99.48	98.39	95.81	97.08	96.43	98.95	97.67	98.10	98.08	98.08	98.08
T14	98.96	99.48	99.22	98.45	99.48	98.96	100.00	98.43	99.21	99.13	99.13	99.13	99.13
T15	99.47	98.43	98.95	97.45	100.00	98.71	100.00	98.43	99.21	98.97	98.95	98.95	98.95
T16	99.48	99.48	99.48	97.93	98.95	98.44	98.94	97.91	98.42	98.78	98.78	98.78	98.78
Macro avg	99.07	98.17	98.61	98.09	98.69	98.38	98.16	98.43	98.29	98.44	98.43	98.43	98.43

## Data Availability

The data used in this study are available from two primary sources. The first dataset is the Bearing Data Center from the Case School of Engineering at Case Western Reserve University, which is accessible online at https://engineering.case.edu/bearingdatacenter, which was accessed on 4 November 2024. The second dataset was provided by Lessmeier et al., entitled Condition Monitoring of Bearing Damage in Electromechanical Drive Systems Using Motor Current Signals of Electric Motors: A Benchmark Data Set for Data-Driven Classification [38].
